# In adult dogs is supplementary tryptophan in the diet effective in reducing signs of anxiety?

**DOI:** 10.18849/ve.v9i4.686

**Published:** 2024-10-24

**Authors:** Lesca Monica Sofyan

**Affiliations:** Colyton Veterinary Hospital, Australia

**Keywords:** anxiety, anxious behaviour, behaviour, canines, dogs, paw complete calm, tryptophan

## Abstract

**PICO question:**

In adult dogs, is dietary supplementation with tryptophan compared with no dietary tryptophan supplement effective in reducing signs of anxiety?

**Clinical bottom line:**

**Category of research:**

Treatment.

**Number and type of study designs reviewed:**

Two studies were found to be considered appropriate in the level of hierarchy of evidence pyramid. One study was a randomised double-blinded, placebo-controlled study and the other was a single-blind, placebo-controlled study.

**Strength of evidence:**

Moderate.

**Outcomes reported:**

One study found no overall significant influence of tryptophan in the diet as an aid in reducing anxiety and fear-related behaviour in anxious dogs in household conditions. In contrast, the second study reported lower anxiety-related behaviour in dogs by owners but did not find significant differences in cortisol levels based on urine cortisol-to-creatinine ratio.

**Conclusion:**

Current evidence reveals two studies that have evaluated the effectiveness of tryptophan as a supplement to the diets of anxious canines for pet owners. One study conducted did not find a significant effect or interaction in reducing anxiety-related behaviour whilst the second study reported lower anxiety-related behaviour in dogs by owners but did not find significant differences in cortisol levels based on urine cortisol-to-creatinine ratios. The use of tryptophan as a supplementary component in the diet is thus not enough as a sole treatment to assist in reducing anxiety in anxious adult dogs.

## How to apply this evidence in practice

The application of evidence into practice should take into account multiple factors, not limited to: individual clinical expertise, patient’s circumstances and owners’ values, country, location or clinic where you work, the individual case in front of you, the availability of therapies and resources.

Knowledge Summaries are a resource to help reinforce or inform decision-making. They do not override the responsibility or judgement of the practitioner to do what is best for the animal in their care.

## Clinical scenario

You are presented with a 1-year-old female neutered Border Collie for a behavioural consultation. During this consultation, the owner is seeking to trial natural-based treatments rather than anxiety medications. The owner mentions a daily chewable that is meant to assist in reducing anxiety long term for anxious dogs. The main ingredient of the product is tryptophan. The owner asks if there is evidence of the efficacy and success in using this long term for reducing anxiety for his pet.

## The evidence

Two studies were found to provide moderate evidence in assessing the effect of tryptophan in the diet in reducing anxiety-related behaviour (Bosh et al., 2009 and Kato et al., 2012). Both studies provided a low risk of bias and moderate evidence in that they were placebo-controlled, and treatment allocation was randomized to the candidates involved.

## Summary of the evidence

**Bosch et al. (2009):** 

Population	**Criteria for eligibility**: Dogs over 6 months of age. Dogs that fitted into the category of anxious behaviour based on a questionnaire derived from the Canine Behavioral Assessment and Research Questionnaire (CBARQ). Criteria for exclusion: Pregnant dogs. Dogs that suffered from, or had, a medical condition receiving behavioural therapy.
Sample Size	207 dogs.
Intervention Details	Random allocation into three treatment groups: **Control diet **(n = 66): The diet constituted of high-protein food brand Casa-Fera Adult Dog Food. **Tryptophan diet** (n = 72): The diet constituted of high-protein dog food brand Casa-Fera Adult Dog Food supplemented with tryptophan. **Fortified diet **(n = 69): The diet constituted of high-protein dog food brand Casa-Fera Adult Dog Food supplemented with tryptophan in combination with beet pulp, salmon oil, soy lecithin, and green tea extract. Questionnaire: An online questionnaire was filled out by the owners based on their dog’s behaviour in their home before the onset of dietary treatment and after 4 and 8 weeks from feeding the diet. The specific questions focused on expressing the degree of stranger-directed fear (3 questions), non-social fear (6 questions), separation-related behaviour (8 questions), attachment or attention seeking behaviour (6 questions), excitability (6 questions) and pain sensitivity (3 questions). Answers were presented on a 5-point scale and awarded a value from 0 to 4. Scores of 0 indicated the behaviour was not performed. Scores of 1 to 3 indicated mild-to-moderate signs of the behaviour. Scores of 4 indicated the behaviour was considered excessive. Lower score values were therefore more favourable. Time Frame for Assessment of Outcomes: The web-based questionnaire to be reported by owners based on their dog’s behaviour was filled out before the onset of dietary treatment and after 4 and 8 weeks of feeding the diet. The controlled open field test was conducted before and after 8 weeks of the dietary treatment. Saliva collection for measurement of cortisol was collected before the onset of diet treatment and 8 weeks after diet treatment. Controlled open-field test: Dogs were left alone and undisturbed for 1 minute in an empty room with background white noise played (novel phase) after which the background noise was switched off (sudden silence phase) for 1 minute. A sound pulse was produced and a novel object (plastic tubing) was dropped from the ceiling. After 1 minute, dogs were led to the test area with their owners. The test area replicated a living room. The owners and the dog were together for 3 minutes then the owner would leave the dog alone for 5 minutes as they would in practical Video recording of behavioural parameters that revealed walking, standing, sitting, lying, high posture, neutral posture, low posture, very low posture, tail wagging, trembling, being close to the novel object, barking, howling, growling, whining, snout-licking, tongue out, paw lifting, sudden sniffing, body shaking, auto grooming, yawning, stretching, freezing, turning head away, urinating or defecating in the environment. The behaviour tests involved a 4-min composite open-field test followed by an 11-min owner separation test. Saliva sampling: 34/66 dogs from the control group and 39/72 from the tryptophan group were selected for saliva sampling. The number of dogs from the fortified trial was not provided. Two saliva samples were taken from the dog’s cheek pouches – the first was before the onset of the open field test and the other was immediately after the open field test. The buccal swabs were performed at 45–60 seconds and stored in Salivette tubes on ice until the end of the day. The tubes were centrifuged at 3000 x g for 10 minutes at room temperature. The resulting saliva was stored at -20 C° at 8 C° until analysis.
Study design	Randomised double-blinded, placebo-controlled study.
Outcome studied	Assess level of anxiety-related behaviour in home-situations via a questionnaire for owners derived from the Canine Behavioral Assessment and Research Questionnaire (CBARQ) and in a controlled open-field test.
Main findings (relevant to PICO question)	Comparing the control diet and tryptophan diet group, the effect of tryptophan in the diet did not significantly affect anxiety-related behavioural parameters derived from the questionnaire. Owners provided lower scores in weeks 4 and/or 8 of the study compared to the initial scores in week 0 (P < 0.05) for the behaviour parameters tested, except for stranger-directed fear, thus indicating a potential reduction in anxiety. For non-social fear, owners awarded significantly higher scores in week 4 than in week 8. There was no overall significant effect or interaction on the influence of dietary treatment on the behaviour of dogs in the controlled open-field test. Weak indications for the dietary effects were found for low body posture (P = 0.087) and body shaking (P = 0.098).
Limitations	Loss of follow-up in some participants. Out of a total of 378 dog questionnaires that were received, only 271 were selected. 51 dogs did not start dietary treatment and 13 dogs were lost to follow-up during the study. Temporal bias due to possibility of dietary or housing changes.

Table note...

**Kato et al. (2012):** 

Population	Criteria for eligibility: Anxious dogs that were determined and scored accordingly based on results from the Canine Behavioral Assessment and Research Questionnaire (C-BARQ). Privately owned dogs. Criteria for exclusion: Dogs under 1 year of age. Pregnant dogs. Dogs that suffered from a medical condition. Dogs that received or were receiving behavioural therapy. Dogs that received or had psychoactive medication.
Sample Size	44 dogs.
Intervention Details	Allocation to two different diets: The study diet (Royal Canin Calm Canine) and control diet (Royal Canin Select Skin Care Vets Plan) for each canine was packaged in white paper bags labelled ‘Food A’ and ‘Food B’ so the content of each diet was blinded to the dog owner throughout the study. The Royal Canin Calm Canine constituted of supplements alpha-casozepine and L-tryptophan. Duration of Dietary Intake: Each diet was administered for 8 weeks with a transitional period of 1 week between diets. Urine Sampling: After 7 weeks of feeding the diet 2 urine samples from the patient were collected and stored at -20 C° until analysis to measure urine cortisol-to-creatinine ratio (UCCR). One sample was obtained 2 hours after leaving the veterinary practice to measure the post-stressor UCCR and another sample was obtained whilst the patient was in its home environment without the occurrence of any special event of stressful condition by the owners. The samples required to be obtained by the owners was collected at the same time of the day to account for any potential circadian rhythm. Analysis of cortisol concentration was measured by using the Cortisol Enzyme Immunoassay Kit and determined by the Jaffe kinetic method. Canine Behavioral Assessment and Research Questionnaire (C-BARQs): Owners completed the C-BARQ after 7 weeks on their allocated diet.
Study design	Single-blind, placebo-controlled study.
Outcome studied	Evaluate the effect of the study diet (Royal Canin Calm Canine constituted with supplements alpha-casozepine and *L*-tryptophan) on the stress response of dogs in stressful situations such as a visit to a veterinary practice and overall effect on anxiety-related behaviours.
Main findings (relevant to PICO question)	Only 28/44 dogs completed the study. Owners gave significantly lower and favourable scores in the study diet compared to the control diet (P < 0.05) based off the C-BARQ. Basal UCCR results was not significantly different in dogs fed either diets (P = 0.26). Post-stressor UCCR in both diets were significantly higher than the basal UCCR (P < 0.01).
Limitations	16 dogs did not complete the study for the following reasons – 3 dogs did not end up starting the dietary treatment, 5 were withdrawn for health reasons not associated with the diet, 4 were withdrawn for reasons associating with moving, hospitalisation of the dog owner and personal reasons and 4 were lost to follow-up during the study.

Table note...

## Appraisal, application and reflection

The C-BARQ questionnaire was developed by researchers at the Centre for the Interaction of Animals and Society of the University of Pennsylvania. The questionnaire consisted of questions to determine the extent of stranger-directed aggression, owner-directed aggression, stranger-directed fear, non-social fear and touch sensitivity. Owners were asked to score their dog’s behaviour from a scale of 0 to 4 rating where 0 = no signs of the behaviour, 1 to 3 = mild-to-moderate signs of the behaviour and 4 = severe signs of the behaviour. The study by Bosch et al. (2009) revealed dietary tryptophan did not have a significant impact on anxious behaviour in privately owned dogs based on a controlled open-field test evaluating anxiety related behaviour and the C-BARQ questionnaire from owners. On the other hand, Kato et al. (2012) found that the supplementation of casozepine and/or tryptophan in the diet may have acted as a determining factor in reducing stress and anxiety-related behaviour as owners gave significantly lower and favourable scores on the C-BARQ questionnaire.

Although the study by Bosch et al. (2009) comprised of favourable research components such as randomisation and blinding of participants of the treatment they were allocated to, the study also possessed a few limitations. External validity is defined as to whether casual relationships may be generalised and applicable to different measures (Steckler et al., 2008). Internal validity in contrast represents whether the variables operated adequately represent the theories and hypothesis proposed (Steckler et al., 2008). Whilst it is useful to know if supplementary tryptophan in the diet assists in reducing anxious behaviour, the treatment options tested should be validated and applicable to the general canine population that exhibit anxious behaviour. This can however be difficult to apply in regard to anxious dogs as every patient is different to one another, and the precursors or triggers of anxiety that a patient is exposed to may be different in current and everyday circumstances.

Appropriate sample sizes are essential in providing a true representation of an underlying population and ensuring that the clinical question proposed is statistically adequate and satisfied (Nayak, 2010). Small sample sizes may not be sufficient to detect a true difference resulting in a false negative (Nayak, 2010). The unequal number of patients amongst each treatment group may have potentially shifted and statistically favoured the effects of tryptophan in the diet therefore, distorting an equal and fair representation.

Clients may often attempt to seek natural-therapeutic remedies or treatment in aid of reducing anxiety-related behaviour in their dogs. Tryptophan is the precursor of the synthesis of the neurotransmitter serotonin and it has been relatively known to decrease serotonergic activity in anxiety and fear related behaviour (Hoyer et al., 2002; Graeff et al., 1996). Tryptophan is found as either a sole or additional dietary component in a wide range of food diets aimed at reducing anxiety and increasing calmness in dogs. DeNapoli et al. (2000) is another study that found the addition of tryptophan supplementation in a low protein diet may be helpful in reducing territorial aggression as behavioural scores were lower in dogs fed a low protein without tryptophan supplement by dog owners.

Current evidence reveals only two studies that have evaluated the effectiveness of tryptophan as a supplement to the diets of anxious canines for pet owners. The resulting outcome of each study differed from one another where Bosh et al. (2009) found no significant influence of tryptophan in the diet in reducing anxiety and fear related behaviour in anxious dogs and Kosh et al. (2012) found owners reported lower anxious-related behaviour in dogs. Despite the differing results, there is moderate evidence to suggest the supplementation of tryptophan in the diet is effective in reducing anxiety-related behaviour. The use of tryptophan as a supplementary component in the diet may be considered as an adjunctive treatment in reducing anxiety in anxious adult dogs.

## Methodology

**Search strategy:** 

Databases searched and dates covered	CAB Abstract database via Web of Science 1973–2024 PubMed database accessed via the NCBI platform 1910–2024
Search terms	anxious OR anxiety) AND (tryptophan OR tryphtophan) AND (dogs OR dog OR canine OR canines)
Dates searches performed:	17 Jun 2024

Table note placeholder

**Exclusion / inclusion criteria:** 

Exclusion	Articles not written in English. Case reports. Case studies. Book chapters.
Inclusion	Meta-analysis. Randomised controlled study. Clinical studies. Cohort studies.

Table note placeholder

**Search outcome:** 

Database	Number of results	Excluded – Articles not written in English	Excluded – Did not relate directly to the factors of the PICO	Excluded – Case reports and studies	Excluded – Book chapters, conference proceedings	Total relevant papers
CABAbstracts	10	0	10	0	0	0
PubMed	10	2	0	0	6	2
Total relevant papers when duplicates removed						2

Table note placeholder

## Acknowledgements

The author would like to greatly thank William Smith, Bridget Sheppard, Jennifer Morris, Clare Boulton, the reviewers, and the team of *Veterinary Evidence* for providing this opportunity. Lastly, but not least, the author also dedicates this paper to Elie Jason Sirrie and Border Collie Cleo Sirrie to hopefully help future patients alike.

## ORCiD

Lesca Monica Sofyan: https://orcid.org/0000-0002-7824-6604

## Conflict of interest

The author declares no conflicts of interest.

## Disclaimer

Knowledge Summaries are a peer-reviewed article type which aims to answer a clinical question based on the best available current evidence. It does not override the responsibility of the practitioner. Informed decisions should be made by considering such factors as individual clinical expertise and judgement along with patient’s circumstances and owners’ values. Knowledge Summaries are a resource to help inform and any opinions expressed within the Knowledge Summaries are the author’s own and do not necessarily reflect the view of the RCVS Knowledge. Authors are responsible for the accuracy of the content. While the Editor and Publisher believe that all content herein are in accord with current recommendations and practice at the time of publication, they accept no legal responsibility for any errors or omissions, and make no warranty, express or implied, with respect to material contained within. For further information please refer to our Terms of Use.
